# Supplementary material for: Feasibility of integrating canine olfaction with chemical and microbial profiling of urine to detect lethal prostate cancer
Source: PLoS One. 2021 Feb 17;16(2):e0245530. doi: 10.1371/journal.pone.0245530 (PMC7888653; doi:10.1371/journal.pone.0245530)
Supplement: S1 Table — (DOCX) [file pone.0245530.s001.docx]

**S1 Table. Sample use among canine olfaction, GC-MS/ANN, and microbial profiling.**

| **Sample ID** | **Age** | **PSA** | **Status** | **Dog Training** | **Dog Trial** | **GC-MS/ ANN** | **Microbiome** |
| --- | --- | --- | --- | --- | --- | --- | --- |
| AWP-5568 | 60 | 3.3 | Control |  |  |  |  |
| AWP-5577 | 45 | 1.6 | Control |  |  |  |  |
| AWP-5585 | 51 | 10 | Control |  |  |  |  |
| AWP-5648 | 59 | 3.56 | Control |  |  |  |  |
| AWP-5679 | 51 | 1.1 | Control |  |  |  |  |
| AWP-5726 | 53 | 6.5 | Control |  |  |  |  |
| AWP-5742 | 79 | 6 | Control |  |  |  |  |
| AWP-5767 | 78 | 2.86 | Control |  |  |  |  |
| AWP-5936 | 64 | 17 | Control |  |  |  |  |
| AWP-5937 | 51 | 12 | Control |  |  |  |  |
| AWP-5986 | 69 | 10.8 | Control |  |  |  |  |
| AWP-6147 | 57 | 4.9 | Control |  |  |  |  |
| AWP-6195 | 55 | 1.2 | Control |  |  |  |  |
| AWP-6201 | 70 | 1.9 | Control |  |  |  |  |
| AWP-6241 | 52 | 10 | Control |  |  |  |  |
| AWP-6255 | 56 | 4.48 | Control |  |  |  |  |
| AWP-6258 | 57 | 4.8 | Control |  |  |  |  |
| AWP-6598 | 80 | 14.2 | Control |  |  |  |  |
| AWP-6651 | 67 | 7.5 | Control |  |  |  |  |
| AWP-8135 | 63 | 3.9 | Control |  |  |  |  |
| AWP-8426 | 63 | 3.4 | Control |  |  |  |  |
| AWP-8926 | 51 | 2.9 | Control |  |  |  |  |
| AWP-9211 | 63 | 4.2 | Control |  |  |  |  |
| JHBUI-1100 | 65 | 4.5 | Control |  |  |  |  |
| JHBUI-1980 | 56 | 3.9 | Control |  |  |  |  |
| JHBUI-2181 | 53 | 3.5 | Control |  |  |  |  |
| JHBUI-2510 | 60 | 3.4 | Control |  |  |  |  |
| JHBUI-2980 | 56 | 8.1 | Control |  |  |  |  |
| JHBUI-3039 | 54 | 6.4 | Control |  |  |  |  |
| JHBUI-3061 | 50 | 4.36 | Control |  |  |  |  |
| JHBUI-3179 | 62 | 4.7 | Control |  |  |  |  |
| JHBUI-3255 | 55 | 18.4 | Control |  |  |  |  |
| JHBUI-3381 | 58 | 14.7 | Control |  |  |  |  |
| JHBUI-3420 | 62 | 5.1 | Control |  |  |  |  |
| JHBUI-3422 | 67 | 4.68 | Control |  |  |  |  |
| JHBUI-3631 | 56 | 5.5 | Control |  |  |  |  |
| JHBUI-620 | 73 | Unknown | Control |  |  |  |  |
| JHBUI-735 | 59 | 6.3 | Control |  |  |  |  |
| AWP-5734 | 60 | 5.1 | Cancer |  |  |  |  |
| AWP-6373 | 50 | 4.8 | Cancer |  |  |  |  |
| AWP-9307 | 66 | 7.3 | Cancer |  |  |  |  |
| AWP-9472 | 72 | 8.6 | Cancer |  |  |  |  |
| AWP-9582 | 71 | 3 | Cancer |  |  |  |  |
| JHBUI-1028 | 71 | 53.4 | Cancer |  |  |  |  |
| JHBUI-2175 | 75 | 4.97 | Cancer |  |  |  |  |
| JHBUI-2719 | 73 | 9.27 | Cancer |  |  |  |  |
| JHBUI-2976 | 64 | 76.8 | Cancer |  |  |  |  |
| JHBUI-2978 | 54 | 23.8 | Cancer |  |  |  |  |
| JHBUI-3147 | 49 | 9.2 | Cancer |  |  |  |  |
| JHBUI-887 | 65 | 6.1 | Cancer |  |  |  |  |
|  |  | **Total Controls** |  | **15** | **21** | **30** | **37** |
|  |  | **Total Cancers** |  | **5** | **7** | **6** | **12** |
