# Supplementary material for: Feasibility of integrating canine olfaction with chemical and microbial profiling of urine to detect lethal prostate cancer
Source: PLoS One. 2021 Feb 17;16(2):e0245530. doi: 10.1371/journal.pone.0245530 (PMC7888653; doi:10.1371/journal.pone.0245530)
Supplement: S3 Table — (DOCX) [file pone.0245530.s003.docx]

**S3 Table. Instrument parameters for HS GC-MS testing of urine samples.**

| **Headspace Conditions** | |
| --- | --- |
| Temperature Zones | |
| Oven Temperature (°C) | 90 |
| Loop Temperature (°C) | 110 |
| Tr. Line Temperature (°C) | 130 |
| Timing | |
| GC Cycle Time (min) | 42 |
| Vial Eq. Time (min) | 60.00 |
| Pressurization Time (min) | 0.15 |
| Loop Fill Time (min) | 0.15 |
| Loop Eq. Time (min) | 0.05 |
| Inject Time (min) | 1.00 |
| Other | |
| Vial Pressure (psi) | 25 |
| Shake | LOW |
| Vial Size (mL) | 20 |
|  |  |
| **GC Conditions** | |
| Zebron ZB-624 | 30 m x 0.25 mm x 1.4 µm |
| Inlet Temperature (°C) | 250 |
| Septum Pure Flow (mL/min) | 3 |
| Mode | SPLIT |
| Split Ratio | 2:1 |
| Column Flow (mL/min) | 1 |
| Oven Program | Hold 40°C for 3 min, ramp 20°C/min to 240°C |
| Total Run Time (min) | 13 |
| **MS Conditions** | |
| Transfer Line Temperature (°C) | 240 |
| Detection Mode | Scan (m/z 30-350) |
