# Supplementary material for: Feasibility of integrating canine olfaction with chemical and microbial profiling of urine to detect lethal prostate cancer
Source: PLoS One. 2021 Feb 17;16(2):e0245530. doi: 10.1371/journal.pone.0245530 (PMC7888653; doi:10.1371/journal.pone.0245530)
Supplement: S4 Table — (DOCX) [file pone.0245530.s004.docx]

**S4 Table. Common contaminant genera in microbiome analyses.**

| Achromobacter | Curtobacterium | Pelomonas |
| --- | --- | --- |
| Acidovorax | Geobacillus | Propionibacterium |
| Agrobacterium | Halomonas | Ralstonia |
| Anoxybacillus | Halorhodospira | Rhodobacter |
| Aquabacter | Herbaspirillum | Shewanella |
| Aquabacterium | Janthinobacterium | Sphingobacterium |
| Aquimonas | Mesorhizobium | Sphingobium |
| Bradyrhizobium | Methylobacillus | Sphingomonas |
| Caulobacter | Methylobacterium | Undibacterium |
| Comamonas | Methylomarinum | Xanthomonas |
| Cryobacterium | Paenibacillus | Diaphorobacter |
| Cupriavidus | Pantoea |  |
