# Supplementary material for: Feasibility of integrating canine olfaction with chemical and microbial profiling of urine to detect lethal prostate cancer
Source: PLoS One. 2021 Feb 17;16(2):e0245530. doi: 10.1371/journal.pone.0245530 (PMC7888653; doi:10.1371/journal.pone.0245530)
Supplement: Attachment — Submitted filename: Response to Reviewers.docx [file pone.0245530.s043.docx]

MASSACHUSETTS INSTITUTE OF TECHNOLOGY	**Andreas Mershin, PhD**

Research Scientist	Tel: +1 (617) 515-4192

Director, Label Free Research Group	Fax: +1 (617) 253-7035

*Center for Bits and Atoms E15-401g*	E-Mail: [mershin@mit.edu](mailto:mershin@mit.edu)

*20 Ames St. Cambridge, MA 02139, USA*	Web: [www.mershin.org](https://www.mershin.org/)

Nov 9 2020

Response to Reviewers/Rebuttal Letter for: “Feasibility of Integrating Canine Olfaction with Chemical and Microbial Profiling of Urine to Detect Lethal Prostate Cancer”

Dear Dr. Culig,

We are thrilled to receive the positive feedback and thoughtful comments of the reviewers on our manuscript. We are indebted to their and your efforts and, as requested have carefully addressed each point as summarized below and have uploaded the marked-up and unmarked versions of the edited manuscript.

1. We have ensured the manuscript meets PLOS ONE’s style requirements including for file naming.
2. Additional details regarding participant consent have been added to the methods section. Specifically, we have clarified that: *“US-originated urine samples used in the final canine training and all GC-MS and canine testing, were obtained under a Johns Hopkins University (JHU) Medicine Institutional Review Board (IRB) approved protocol with written informed consent. For early training and canine testing the UK-originated samples were collected from participants identified by a member of the clinical team who provided them a verbal explanation and information sheet outlining the research. Participants then provided verbal informed consent, confirmed by a signed consent form and completed health questionnaire.”*
3. We confirm the following proposed statements regarding the funding statement and competing interest statement are accurate and suitable to appear alongside the manuscript.

3.1&3.2 We confirm the following proposed statement is accurate and suitable to appear alongside the manuscript. We have updated the author roles in the Author Contributions section of the online submission form and have included an amended Funding Statement as follows: *"This work was funded by the Prostate Cancer Foundation Grant (18PILO02) received by CG, AM, and KS. PCF provided partial salary support for authors CG, RH, PMS, AM, and had a role in the study design and preparation of the manuscript, but had no role in the data collection and analysis or the decision to publish. The National Cancer Institute of the National Institutes of Health provided support for WYL and QG (Award Number SC1CA245675). Imagination Engines, Inc. provided support in the form of salary for ST. The NCI NIH and Imagination Engines, Inc. played a role in study design, analysis, decision to publish, and preparation of the manuscript. The specific roles of these authors are articulated in the ‘author contributions’ section."*

We confirm the following proposed statement is accurate and suitable to appear alongside the manuscript*: "The authors have read the journal’s policy and have the following competing interests: ST is a paid employee of Imagination Engines, Inc. This does not alter our adherence to PLOS ONE policies on sharing data and materials. There are no patents, products in development or marketed products associated with this research to declare.”*

4. We have added the corresponding author’s (AM) ORCID# [0000 0001 8194 6241]
5. The map as well as all other graphics in Figure 1 (i.e. the USA & UK maps and flags, beaker and test tubes) were created by MIT Center for Bits and Atoms student, Alfonso Para Rubio. An acknowledgment of thanks for Alfonso has been added to the manuscript.

Reviewer #1’s comment 1:

“ Several markers and tests have been established (e.g. Prostate Health Index (phi), SelectMDx, PCA3) the last years. The authors should discuss and compare their findings with other biomarkers and tests.”

In response to this comment we have now added the required discussion to the text:

*The purpose of this study was to demonstrate the feasibility of integrating the analysis of prostate cancer patient urine samples using canine olfaction, with chemical and microbial profiling.  Diagnostic tests beyond PSA have been established in the last few years and are relevant to the planned second phase of this study which will aim to compare the receiver operating curves of integrating canine olfaction and chemical and microbial profiling with diagnostic tests such as Prostate Health Index (phi), SelectMDx, and PCA3. As in the case of PSA, these are specific molecular ID biomarker-driven diagnostic metrics while the olfactory approach central to our study depends on the emergent property of scent character. The olfactory diagnostic signal is not necessarily bound to an immutable (set of) molecular identities(s) as the same functional scent character can be conferred to the dogs and machine olfactors via a variety of VOC cocktails. We do note that biomarker-driven as well as emergent-property powered diagnostic metrics, examined collectively, are reasonably expected to paint a more accurate picture of the condition and is likely that their usefulness in directing intervention decisions can be enhanced with the addition of a microbial profiling diagnostic examined here. This integrative approach is a focal point for our future research direction.*

Reviewer #1’s comment 2:

*“The authors should discuss if their “VOC signature” presented in this study correlates with signatures identified in other cancers to distinguish between a PCa signature and a general cancer signature”*

In response to reviewer’s comment 2 we have added the following to the discussion:

*Much work has been done in the past by groups including our own to determine a specific set of VOC signature biomarkers (by name and potentially relative or absolute concentration). Efforts to mine the volatilome for robust molecular species signatures indicating a number of conditions including prostate [46], colorectal [48], liver [49], and lung cancers [48] have consistently yielded schemes where a set of selection rules can be determined cleaving any sufficiently rich data set into positive and negative control clusters. The emergent issue is that just as seen with canines, it appears that no unique (set of) molecule(s) can be found as unambiguous biomarkers and instead it is the scent character, as opposed to the molecular composition of scent that communicates the cancer signature to canines. GC-MS produces a list of VOCs that carry the information for each experimental case but such lists themselves unfortunately do not generalize to provide a “master biomarker template” in the form of molecular species identifications and concentrations [48]. This shows the potential for prostate cancer diagnoses, but validation of the VOCs remains as future research objectives.*

Sincerely, on behalf the co-authors

Andreas Mershin, Ph.D.
